# Recent Progress of Molecularly Imprinted Technique for the Detection of Mycotoxins in Food

**DOI:** 10.3390/foods13244125

**Published:** 2024-12-20

**Authors:** Yuan Wang, Dizhe Wei, Yu Wang, Meng Wang, Wenlei Zhai

**Affiliations:** 1 Institute of Quality Standard and Testing Technology, Beijing Academy of Agriculture and Forestry Sciences, Beijing 100097, China; 20233695@stu.sxau.edu.cn (Y.W.); weidz@iqstt.cn (D.W.); 2 School of Food Science and Engineering, Shanxi Agricultural University, Taiyuan 030801, China; sxtgwy@126.com

**Keywords:** molecularly imprinted polymers, mycotoxins, food safety, surface-enhanced Raman spectroscopy, solid-phase extraction, fluorescence sensors, electrochemical sensors, surface plasmon resonance

## Abstract

Mycotoxins are a group of toxic metabolites produced by fungi that infect agricultural products. Consuming mycotoxin-contaminated foods and feeds can cause various adverse health effects in humans and animals. Therefore, developing rapid and sensitive analytical methods for detecting mycotoxins is an urgent task. The molecularly imprinted technique is an advanced analytical tool for the specific recognition and selective enrichment of target molecules. For the development of rapid detection methods for mycotoxins, synthesized molecularly imprinted polymers (MIPs) can serve as specific recognition elements. By integrating MIPs with various sensing platforms, such as solid-phase extraction, electrochemical sensors, fluorescence sensors, surface-enhanced Raman spectroscopy, and surface plasmonic resonance sensors, remarkable progress has been made in the detection of mycotoxins in foods. This review focuses on the advances in the application of MIPs for the rapid detection of various mycotoxins over the past five years. The development of new MIP synthesis methods is categorized and summarized. Moreover, the future potential of MIP-based methods for mycotoxin detection is also discussed and highlighted.

## 1. Introduction

Food safety has been one of the major concerns for public health globally. As one of the major contaminants in agricultural products and food, mycotoxins are secondary metabolites produced by fungal pathogens under specific environmental conditions [1]. To date, more than 400 forms of mycotoxin compounds have been identified, primarily generated by fungi such as *Aspergillum*, *Penicillium*, *Fusarium*, and *Alternaria*. Common mycotoxins include aflatoxins (AFs), ochratoxin A (OTA), zearalenone (ZEN), deoxynivalenol (DON), fumonisins (FMs), and patulin (PAT) [2,3,4]. Mycotoxin contamination commonly occurs in a wide range of food products, including cereals, nuts, tea, and plant-based beverages [5]. The route of contamination can occur during crop cultivation, storage, and food production processes. The accumulation of mycotoxins is exacerbated by mold growth due to global warming and extreme climatic events [5,6,7,8]. Mycotoxicosis can result from exposure to a single mycotoxin or a combination of mycotoxins, which can have chronic and acute adverse effects on animals and humans, endangering their health [9,10]. They are mainly absorbed through the gastrointestinal tract and undergo metabolic transformation in the liver, causing various harmful effects [11]. For example, aflatoxin B_1_ (AFB_1_) is a potent carcinogen. It can form adducts with DNA, which can lead to DNA damage and mutations, leading to diseases including liver cancer [12]. OTA can bind to proteins in the kidneys and cause kidney damage [13]. Consequently, many governments and international organizations established strict maximum residue limits (MRLs) for each highly toxic mycotoxin. For example, the Codex Standard, which serves as an international reference through the joint efforts of FAO/WHO, set MRLs of 0.5–15 μg/kg for AFs in cereals, nuts, and milk [4]. Therefore, developing rapid and sensitive analytical methods for the detection of mycotoxins has been an urgent task.

Various instrumental methods and rapid screening techniques have been developed to monitor mycotoxin levels, with gas chromatography–mass spectrometry (GC–MS) and high-performance liquid chromatography–mass spectrometry (HPLC–MS) being used for the detection of mycotoxin residues. Although these analytical methods offer high sensitivity for qualitative and quantitative detection of mycotoxins, they require tedious sample pre-treatment processes, and the sophisticated instruments need professional technicians to operate, making them unsuitable for field testing [14,15,16]. Therefore, rapid screening techniques have been extensively studied and promoted. Among these, specific recognition is essential for the rapid and accurate detection of mycotoxins. Several methods have been developed to detect analytes using specific recognition elements, such as aptamers and antibodies [17]. However, the development of new antibodies and aptamers for target molecules is costly. Furthermore, these biomolecules exhibited limited stability in complex testing matrices, which could result in false negative readouts. In contrast, molecularly imprinted technology (MIT) offers an ideal solution because it can mimic the specific recognition capabilities of biological antibodies while being more stable, cost-effective, and chemically robust. Molecularly imprinted polymers (MIPs) are synthetic materials with tailored cavities that can serve as recognition elements in various sensing platforms, providing an efficient method for identifying target molecules [18,19,20]. Notable advancements have been made in the detection of mycotoxins in food by integrating MIPs with various analytical methods, including electrochemical sensors [21], fluorescence sensors [22], and surface-enhanced Raman spectroscopy (SERS) [23].

**Figure 1:**
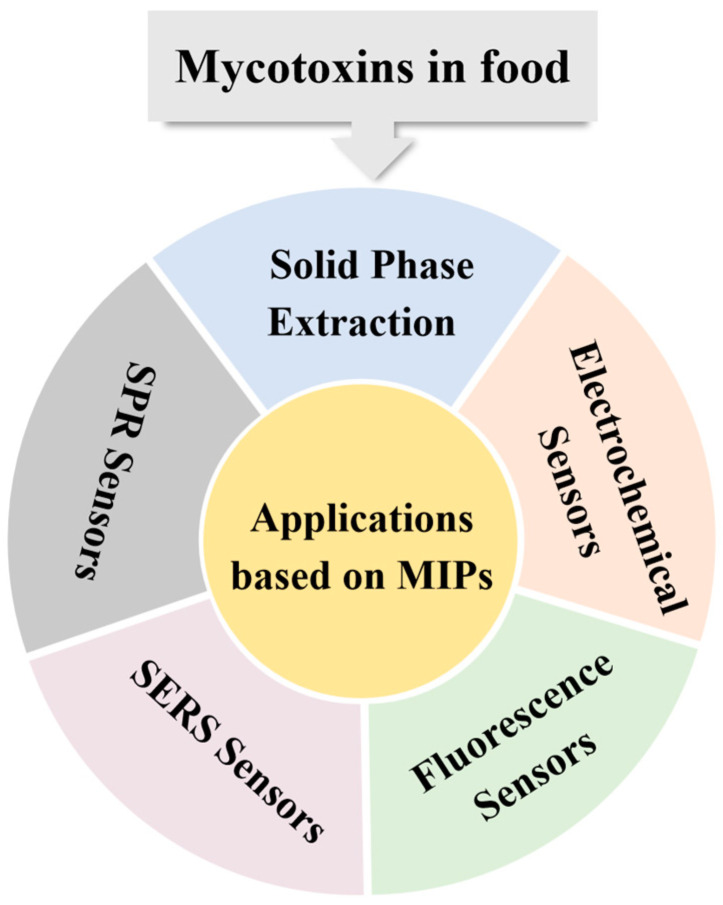
Schematic illustration of MIPs for rapid detection of mycotoxins in foodstuffs.

Several recent reviews focused on the application of MIPs for the selective identification of food contaminants. Cavalera et al. discussed recent advances in the use of MIPs as solid-phase extraction materials for mycotoxin detection, with a particular focus on the application of mimetic molecules in the synthesis of mycotoxin-imprinted materials [24]. Geng et al. [25] reviewed the progress on the combination of MIPs and metal–organic frameworks (MOFs) in the field of food safety detection over the last five years, starting from 2019. Hua et al. [26] focused on the performance of MIP-based sensors, including MIP-based particles and films, and their application in mycotoxin detection. However, comprehensive review focusing on the progress of MIT-based analytical platforms for the detection of mycotoxins in food have not been conducted. Therefore, this review mainly aimed to summarize the recent progress on this topic. We first introduced the preparation methods of MIPs. As shown in Figure 1, the selected studies were categorized into five major sections based on different application areas: solid-phase extraction (SPE), electrochemical sensors, fluorescence sensors, SERS sensors, and surface plasmon resonance (SPR) sensors. The advantages of these tools, using MIPs as recognition elements, and their performance in real sample analysis were highlighted. The current challenges and possible directions for improvement were also discussed.

## 2. Preparation Strategies of MIPs

MIPs are versatile and can be tailored for the detection of sample analytes depending on the target. The basic process of preparing MIPs typically involves pre-polymerization, polymerization, and elution of the template molecules. First, the template molecules interact with suitable functional monomer(s) to for a complex. Then, a polymer network with imprinted templates is generated by initiating a polymerization reaction under the influence of cross-linking agents and initiators [27]. Finally, physical or chemical methods are employed to remove the template molecules. Therefore, three-dimensional polymers with fixed cavities that complement the shape and function of the template molecules for recognition are obtained [28,29,30].

Template molecules, functional monomers, crosslinkers, initiators, and porogen agents are key components in the synthesis of MIPs [31]. Among these, the template molecule plays a crucial role in MIP preparation as it directly affects the specificity and selectivity of the MIPs [32]. Functional monomers are molecular building blocks that form stable intramolecular interactions with the template molecules (e.g., methacrylic acid, sulphonic acid, and acrylic acid). Commonly used monomers typically contain two moieties: the recognition unit and the polymerizable unit. The formation of stable complexes between functional monomers and template molecules via noncovalent interactions is a key step in MIP synthesis. After pre-polymerization, the functional monomers are immobilized around the template molecules through the introduction of a crosslinker, forming a highly rigid cross-linked network [33]. The type and amount of crosslinker influence the morphology, stability, recognition, and binding capability of the final MIPs. Ethylene glycol dimethacrylate (EGDMA) and trimethylolpropane trimethacrylate are among the most popular crosslinkers [31]. MIPs are typically prepared through free radical polymerization, electropolymerization, and photopolymerization. Porogens serve as dispersing media and pore formers in the polymerization process. The types of nonpolar and polar pore-forming agents (e.g., acetonitrile, chloroform, and toluene) [34], commonly used to reduce interference in the pre-polymerization process, affect the imprinting efficiency of MIPs, the affinity between template molecules and functional monomers, and the morphology of the polymers [32,35].

Based on different polymerization methods, MIPs can be classified as bulk polymerization, precipitation polymerization, in situ polymerization, emulsion polymerization, and suspension polymerization [36]. Table 1 summarizes the advantages and shortcomings of different methods for the preparation of MIPs, as well as their application platforms.

### 2.1. Bulk Polymerization

Bulk polymerization is one of the earliest methods used in the preparation of MIPs, aiming to fully imprint the template molecule into the polymer network, which is mixed with functional monomers, crosslinkers, initiators, and undergoes polymerization to form a complete polymer network under appropriate conditions [37]. Upon completion of polymerization, the template molecules are removed by physical or chemical means, leaving behind a cavity structure. The resulting bulk polymers are then dried, crushed, ground, and sieved to obtain MIP powder. The advantages of this method include its ease of operation, no requirement of complex instruments, and its capability of generating additional binding sites, which facilitates the enhancement of adsorption rate [38]. However, bulk polymerization has some drawbacks. First, nonspecific binding sites may be generated during the preparation process, which limits the recognition accuracy of MIPs. For large molecular templates, the diffusion of templates into the inner cavities of MIPs may be notably limited, compromising the sensitivity and selectivity of MIP applications [39]. Finally, the time-consuming process and potentially inhomogeneous particle size and shape compromise the binding sites of the MIPs, thus affecting their performance in applications [40].

### 2.2. Precipitation Polymerization

Precipitation polymerization is an effective method for preparing MIPs and is particularly suitable for the synthesis of spherical beads at the micro and nanometer scale. This method is ideal when MIPs are used as stationary phases in SPE or chromatographic columns. This polymerization method does not require surfactants or other additives and involves polymerizing monomers, crosslinkers, and template molecules in a mixture of excess solvents and porogen agents. As the reaction progresses, the solubility decreases, forming stable polymer particles, while the porogen become encapsulated within the microspheres, creating the pore structure after the completion of polymerization [39]. The advantages of this method include its easy operation and control of the particle size, shape, pore structure, and surface properties of the microspheres by adjusting the polymerization conditions. Moreover, the prepared microspheres have a high degree of crosslinking, which enhances the selectivity and stability of MIPs. However, this polymerization method is time consuming and requires a substantial amount of solvent compared to the bulk polymerization method [41].

### 2.3. In Situ Polymerization

In situ polymerization allows for direct polymerization in a specific environment and is suitable for the preparation of MIPs with specific shapes, sizes, and functions. In this method, template molecules, functional monomers, and crosslinkers are mixed in a specific reaction environment through sonication. By introducing an initiator, the mixture is exposed to heat or light to trigger the polymerization reaction [42], forming polymer nanoparticles and molecules bound to a thermoset polymer nanocomposite [43]. MIPs produced by in situ polymerization exhibit high-quality chemistry and well-defined nanostructures [39], making them applicable for various purposes, such as drug delivery, separation and purification, biosensors, and catalytic applications. Compared to bulk polymerization, in situ polymerization enables direct functionalization of sensing interfaces, avoiding the need for drying and grinding. However, this method requires precise control over the polymerization conditions, such as temperature, pressure, and reaction time, to ensure the desired structure and properties of the polymer.

### 2.4. Emulsion Polymerization

Emulsion polymerization is widely used for preparing MIP nanoparticles with exposed binding sites on their surface [28]. This method involves polymerization in an emulsion system, where surfactants are used to stabilize the emulsion droplets. This method enables the preparation of MIP nanoparticles or microspheres with a uniform distribution of surface-exposed binding sites, offering a high-specific surface area and reusability and effectively improving the binding efficiency and kinetics of target molecules [44]. However, emulsion polymerization generally requires the use of surfactants, which may weaken the binding capacity of MIPs by blocking the binding sites. Additionally, the MIP particles must be washed and purified to remove unreacted reagents after polymerization, increasing the complexity of post-processing [30,34].

### 2.5. Suspension Polymerization

Suspension polymerization is advantageous in overcoming the limitations of low overall process efficiency compared with other polymerization methods [45]. This method requires two stationary phases: the organic stationary phase (monomer, crosslinker, solvent, template, and initiator) and the continuous aqueous stationary phase (surfactant) [40]. Additionally, perfluorocarbon liquids and mineral oils can be used as continuous phases [32], enabling one-step polymerization and the formation of spherical particles with relatively large sizes (ranging from μm to mm). In suspension polymerization, template molecules are relatively easy to release from the polymer, and the one-step polymerization operation is simpler, making it suitable for large-scale applications [45]. Compared to emulsion polymerization, polymerization is less efficient, and the particles it produces are larger in size and less recognizable [39]. Complex processes and the use of surfactants may also contaminate MIPs, and these contaminants are difficult to remove, often interfering with the imprinting process [41].

### 2.6. Surface Imprinting Polymerization

Surface-imprinted polymerization is a polymerization reaction that occurs on the surface of a carrier. During the process, most of the binding sites are located on the outer layer or surface of the polymer. This phenomenon prevents the templates from embedding deeper than conventional polymerization, resulting in higher separation efficiency, faster binding sites, and less “encapsulation”. Additionally, the surface-imprinted layer contains cubic cavities that can complement the target analyte and exhibit specific recognition functions, which enhance the diffusion and binding efficiency between the target molecule and the imprinted site. Surface imprint polymerization is simple, fast, and convenient, making it widely used in applications such as sensors, separation and purification, catalysis, and biomedicine [37,46,47].

## 3. Applications Based on MIPs

### 3.1. Solid-Phase Extraction

For accurate determination of mycotoxins in food, one main challenge is the matrices interference caused by the mixing of mycotoxins with other components [6]. Therefore, effective sample pretreatment is necessary to separate and enrich the target mycotoxins while eliminating matrix interference before analysis. SPE, first introduced in 1970 [48], had a considerable impact on the field of analytical sciences due to its low solvent consumption [49], high enrichment factor, and ease of automation compared to liquid–liquid extraction [33]. The technique is designed to extract and pre-concentrate target compounds from sample matrices by adsorbing the analytes onto suitable solid adsorbents [50]. Molecularly imprinted solid-phase extraction can enhance the selectivity of traditional SPE materials, thus serving as an ideal adsorbent for SPE. A detailed summary of sensing parameters and conditions is included in Table 2. For example, Fan et al. [51] prepared MIPs for the SPE treatment of AFB_1_. They optimized the ratio of monomer *N*-isopropylacrylamide and the dummy template 7-ethoxycoumarin (7-EOC) via molecular simulation and synthesized dummy molecularly imprinted polymers (DMIPs) with good adsorption capacity and selectivity for AFB_1_. Subsequently, DMIPs were applied to SPE to enrich AFB_1_ from peanut samples. The use of analyte mimics as template molecules was not only consistent with the concept of green chemistry due to its reusability, minimization of solid waste generation, and chemical and mechanical stability, but also prevented false positive results due to template leakage [6]. The results show that the method was highly sensitive for the detection of AFB_1_ in peanut samples with a limit of detection (LOD) of 0.1 μg/L, and the method was successfully applied to the analysis of AFB_1_ in peanut samples. In another study, Song et al. [52] used the suspension polymerization method for preparing AFB_1_ MIPs. A biocompatible medium combined with MIPs was prepared using the structural analogue 6-methyl-4-phenylchroman-2-1 as a dummy template and α-methacrylic acid (α-MAA) and glycidyl methacrylate (GMA) as co-monomers. The obtained MIPs were loaded into an SPE column and applied to enrich AFB_1_ from soy sauce samples, achieving a detection limit of 0.05 ng mL^−1^. Rui et al. [53] used mesoporous silica FDU-12 as a carrier and 7-acetoxy-4-methylcoumarin as a pseudo-template to prepare an SPE adsorbent for AFs. The FDU-12@MIP adsorbent was applied to spiked cereal samples, including wheat, rice, and maize, for the detection of aflatoxins G_1_, G_2_, B_1_, and B_2_. The recoveries ranged from 82.6% to 116.7%, with detection limits for aflatoxins G_2_, G_1_, B_2_, and B_1_ determined to be 0.05, 0.06, 0.06, and 0.05 μg/kg, respectively.

**Figure 2:**
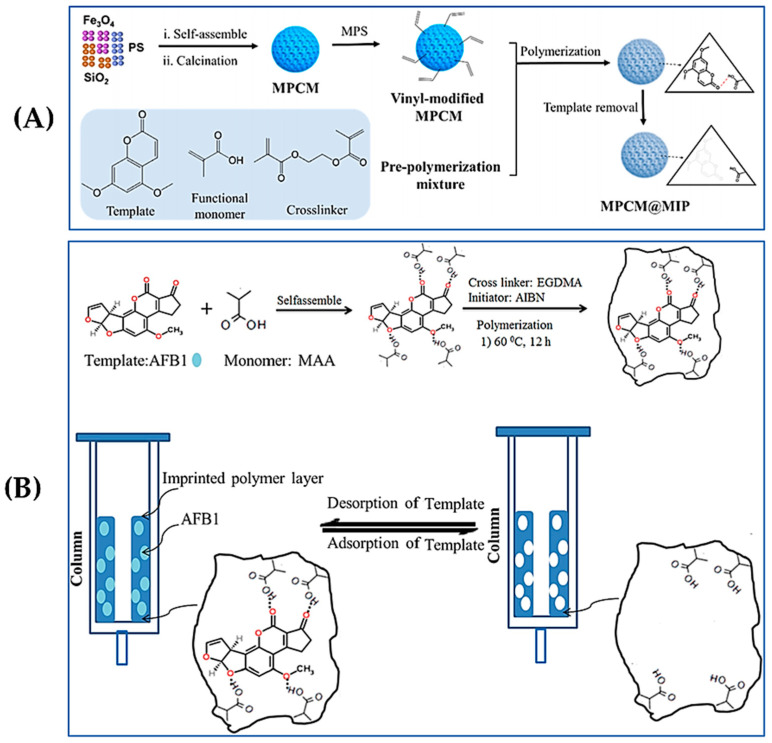
(**A**) Schematic illustration of the preparation of MIPs for molecularly imprinted microspheres with three-dimensionally ordered microporous magnetic reverse photonic crystal microspheres. Reproduced with permission from [55]. Copyright 2022, American Chemical Society. (**B**) Schematic illustration of an immunoaffinity column@MIPs molecularly imprinted polymers as a sorbent for the SPE of AFB_1_. Reproduced with permission from [62]. Copyright 2023, Elsevier.

The introduction of magnetic nanoparticles into the synthesis of MIPs to obtain magnetic MIPs (MMIPs), which endow them with additional magnetic response functionality, also gained considerable attention. MMIPs bind highly selectively to target imprinted molecules and analogues [54]. As shown in Figure 2A, Wang et al. [55] developed a MIP based on a three-dimensional ordered microporous magnetic inverse photonic crystal microsphere for the selective capture of AFB_1_ in soy sauce and vinegar. In this study, 5,7-dimethoxycoumarin was used as the template, and methacrylic acid was employed as the functional monomer. The MIPs were prepared using a microfluidic self-assembly technique, which facilitates rapid separation from the sample solution due to the magnetic properties, simplifies the pre-treatment steps, and improves detection efficiency. When combined with HPLC, a low LOD of 0.4 ng/mL was achieved for AFB_1_. Similarly, for the preparation of MMIPs, Suo et al. [56] synthesized MMIPs using Fe_3_O_4_ nanoparticles as the magnetic core and 5,7-dimethoxycoumarin as a dummy template. These MMIPs were prepared through the self-polymerization of dopamine (DA) in tris (hydroxymethyl) aminomethane hydrochloride (tris-HCl) buffer, which addressed the issue of template leakage [57]. In this study, novel MMIPs based on Fe_3_O_4_@PDA were prepared with high selectivity for AFB_1_ and AFB_2_ in core, peanut, and edible oil samples, which can effectively avoid the interference of other substances in the sample matrix. The maximum adsorption capacity was determined as 0.46 and 0.047 mg/g for AFB_1_ and AFB_2_, respectively, with LODs as low as 0.0024 and 0.0004 ng/mL, respectively, by combining with UPLC–FLD.

Traditional MIPs applied in SPE have limitations such as limited adsorption capacity of the target molecules and low mass transfer rate. To address this issue, MOFs and covalent–organic frameworks (COFs) are widely used in combination with MIPs due to their excellent versatility, recognition properties, and practical applicability. In particular, MOF composites have advantages over other porous materials in terms of mechanical and chemical stability, selectivity, large surface area, scalability, and processability. The mechanical strength, selectivity, and adsorption capacity can be improved through the use of this novel composite of MOF and MIPs [58,59,60,61]. As shown in Figure 2B, Kardani et al. introduced a novel SPE column for the extraction of AFB_1_, AFB_2_, AFG_1_, and AFG_2_ from cereal samples [62]. In this study, MOF-DES@MIPs were prepared by combining MOF, deep eutectic solvent (DES), and MIP for the first time. Benefitting from the tunable pore size and large specific surface areas of MOF, additional active sites were provided for MIPs to improve the adsorption capacity and selectivity. Through the formation of various interactions with the target AFs, including hydrogen bonding, electrostatic interactions, and non-covalent interactions, the adsorption performance of the hybrid material was improved. DES, used as the substrate for MOFs, improved the stability of the MOF particles and extended the lifetime of the SPE columns. The introduction of MIPs endowed the SPE material with specific recognition capability for AFs, effectively reducing interference from non-target compounds. After optimizing the experimental conditions, the prepared SPE columns were coupled with HPLC for the detection of four AFs, with LODs ranging from 0.023 μg/kg to 0.033 μg/kg. COFs are another class of porous framework materials formed through strong covalent bonds [63]. These materials have large specific surface area, high thermal stability, narrow pore size distribution, and low density. Benefitting from these advantages, COFs can also serve as ideal carriers for MIP modifications [60]. Su et al. [64] developed a novel molecularly imprinted flexible covalent–organic framework (MI–FCOF) for the selective identification and efficient extraction of AFs. By introducing flexible units during the synthetic process, MI–FCOF can adapt to the template molecules and form imprinted cavities with high selectivity. In this study, MI–FCOF exhibited an excellent adsorption capacity for AFs, which was three times higher than that of unblotted FCOF. When combined with HPLC, MI–FCOF enabled matrix-free determination of AFs in food samples, with detection limits as low as 0.003–0.09 ng/mL.

### 3.2. Electrochemical Sensors

Electrochemical sensors use electrochemical reactions to convert the presence of the target molecules into detectable electrical signals [65]. The major advantages of electrochemical sensors include ultrahigh sensitivity, fast response, and low cost [66]. These sensors are widely used in environmental monitoring, biomedicine, food industry, and other fields [67]. However, conventional electrochemical sensors are susceptible to food matrix effects [68]. MIPs are promising materials for the development of sensitive membranes for a variety of sensors due to their excellent specificity and environmental tolerance. In these studies, MIPs are immobilized on the electrode surface as the recognition elements. When the target molecules bind to the MIPs, they cause a change in the electrical signal on the surface of the electrode, such as changes in current, voltage, conductivity, capacitance, or impedance. Qualitative and quantitative analysis of mycotoxins in food can be realized by measuring the changes in electrochemical signals [69].

For the MIP assembly process, several commonly used methods modify the electrode surface with synthetic MIP films. In the detection of mycotoxins in foodstuffs [70], most studies involve an electropolymerization process, in which a polymer layer is formed on the electrode surface by electrochemical polymerization in a system containing templates and electroactive functional monomers [71]. Cavities for specific recognition are generated by removing the templates in the electropolymerization medium. Using this strategy, a homogeneous coating can be achieved in a short period of time, offering remarkable advantages over the classical synthetic routes for MIPs. For instance, Chen et al. [72] developed an electrochemical sensor based on biomass-derived porous carbon materials and MIPs for the detection of AFB_1_. This study utilizes Elaeagnus gum as the source to prepare porous carbon materials doped with nitrogen, sulfur, and phosphorus, which are then modified on the surface of a glassy carbon electrode (GCE). Subsequently, an MIP film was formed on the porous carbon surface by in situ electropolymerization of phenol as the monomer and AFB_1_ as the template. AFB_1_ can be determined by measuring the current signal response in differential pulse voltammetry mode. The sensitivity of this sensor can reach 1.7 pM for AFB_1_, and real sample analysis was conducted in cinnamon.

**Figure 3:**
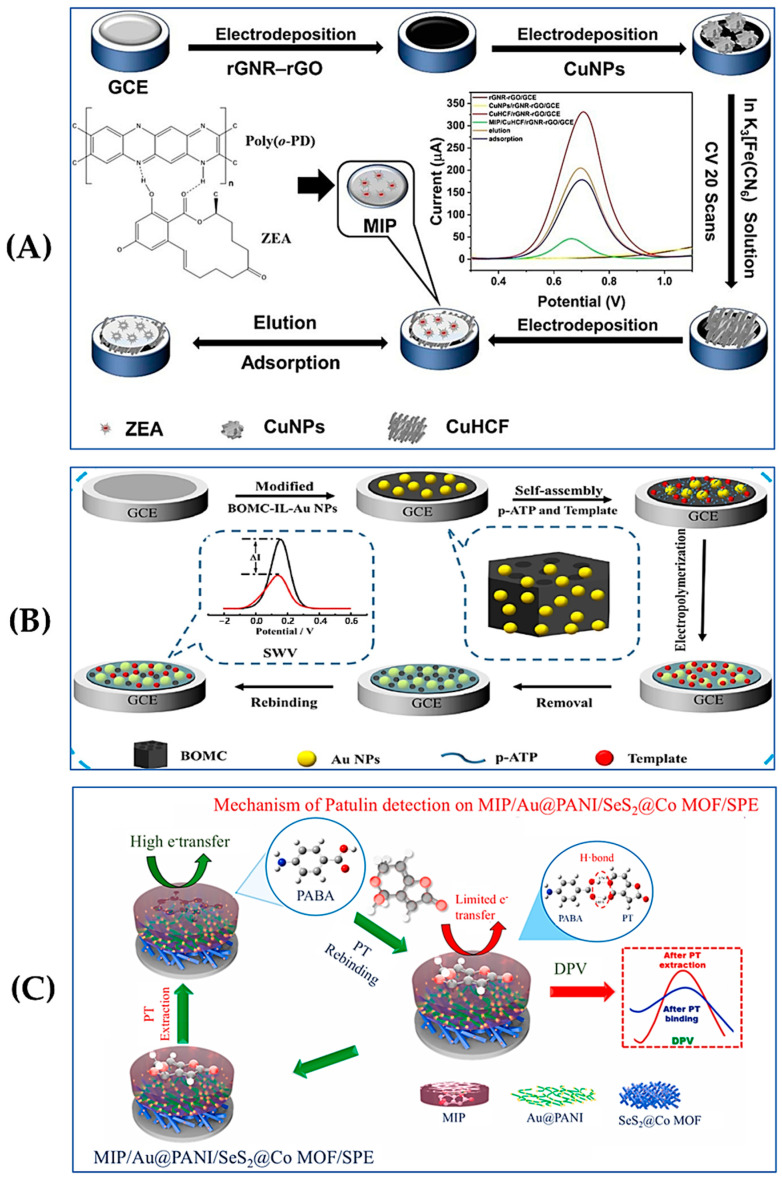
(**A**) Schematic illustration of the construction of MIP/CuHCF/rGNR-rGO/GCE for the detection of ZEN. Reproduced with permission from [74]. Copyright 2024, Elsevier. (**B**) Schematic illustration of the preparation process of MIP/BOMC-IL-Au NPs/GCE. Reproduced with permission from [75]. Copyright 2020, Elsevier. (**C**) Schematic illustration of a MIP/Au@PANI/SeS_2_@Co MOF/screen printed electrode for the detection of PT. Reproduced with permission from [77]. Copyright 2021, Elsevier.

In electrochemical sensors, the detection of analytes in foodstuffs is based on changes in current or voltage at the electrode surface [73]. Thus, common MIPs can only produce weak response due to poor electrical activity. Other conductive materials, such as metal nanoparticles, can be introduced to improve the conductivity of the MIP layer [70]. Zhou et al. recently reported a good example [74]. As shown in Figure 3A, a mixture of reduced graphene nanoribbons and reduced graphene oxide was modified on a GCE using electrochemical deposition to improve the conductivity and active surface area of the electrode. Subsequently, copper nanoparticles and copper hexacyanoferrate were further deposited to enhance the sensitivity and self-reporting capability of the sensor. Finally, MIP membranes were prepared via electropolymerization on the outside layer using ZEN as the template and o-phenylenediamine as the functional monomer. By observing the change in current value, the sensitive detection of ZEN was realized with an LOD of 0.09 ng/mL. Hu et al. used coumarin-3-carboxylic acid [75], a structural analogue of ZEN, as the template, forming a molecularly imprinted film on the electrode surface by electropolymerization with *p*-aminothiophenol (*p*-ATP) as the monomer. As shown in Figure 3B, the electrode deposited gold nanoparticles (Au NPs) on the surface of ionic liquid (IL)-modified boron-doped ordered mesoporous carbon using the hydrothermal method, improving the conductivity and surface area of the electrode and enhancing the sensitivity and stability of the sensor. A LOD of 1 × 10^−4^ ng/mL was achieved in the square-wave voltammetry (SWV) mode. A spiking experiment was also performed using crop, rice, and beer as the real samples. Alternatively, a MIP layer can be directly generated on the surface of conductive materials such as graphene or carbon nanotubes to increase the sensitivity of the sensor. In this case, Rehman et al. [76] developed an electrochemical sensor based on graphene carbon nitride (g-C_3_N_4_) nanosheets modified bovine serum albumin@MnO_2_ (BSA@MnO_2_) nanocomposites and MIPs. The responsiveness to ZEN was improved due to the excellent conductivity and specific surface area of g-C_3_N_4_ and the stability of BSA@MnO_2_. The LOD of ZEN was determined to be 0.25 ng/L under the SWV mode.

In addition to metal nanoparticles and carbon-based conductors such as graphene, the use of MOFs has also been recently explored in the field of electrochemical sensors. As shown in Figure 3C, Selvam et al. [77] used SeS_2_ nanoparticle-loaded Co MOF with Au@PANI nanocomposites to modify the screen-printed electrode. They also screened the suitable monomer p-aminobenzoic acid using density functional theory calculations and introduced MIPs by electropolymerizing the reaction to prepare molecularly imprinted working electrodes, which enhanced the conductivity and stability of the sensor. The results of differential pulse voltammetry detection of patulin show that the optimized LOD was 0.66 pM. Apple juice samples were selected to demonstrate its potential application in the field of food safety.

### 3.3. Fluorescence Sensors

Conventional MIPs show specific recognition sites that selectively adsorb the target molecules. However, they lack the capability to output detection signals. Fluorescence sensors contain signal output units for the application in rapid and on-site detection. Molecularly imprinted fluorescent sensors can be constructed by doping fluorescence components into MIPs, achieving specific recognition. These sensors convert the binding of target molecules and MIPs into a detectable fluorescence signal, which allows highly sensitive and rapid detection [78].

**Figure 4:**
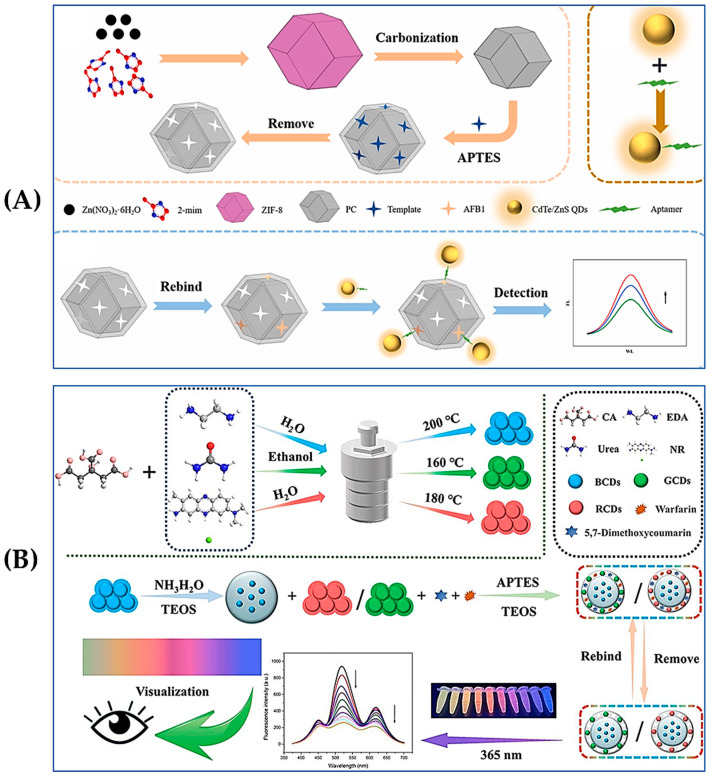
(**A**) Schematic illustration of a fluorometric biosensor based on MIP and CdTe/ZnS-Apt. Reproduced with permission from [81]. Copyright 2023, Elsevier. (**B**) Schematic illustration of a MIP-based ratio metric fluorescence sensor based on three-emission carbon QDs. Reproduced with permission from [84]. Copyright 2023, Elsevier.

For MIP-based fluorescence sensors, the use of suitable fluorescence material is crucial for the performance of sensors. Quantum dots (QDs) are among the most popular nanomaterials for constructing MIP-based fluorescence sensors due to their excellent stability, high quantum yield, broad absorption range, and narrow photoluminescent emissions. However, conventional QDs often contain heavy metal elements that can be harmful to human health. To address this issue, heteroatoms have been incorporated in the synthesis of QDs. Metals such as Mn and Cu are commonly used to prepare doped ZnS QDs because of their advantages, including low self-quenching and strong resistance to thermal and photochemical interference [79]. Chmangui et al. [80] developed a fluorescence probe for the detection of AFs using MnCl_2_-doped ZnS QDs conjugated with MIPs. MIP was synthesized using 5,7-dimethoxycoumarin as the alternative template and methacrylic acid (MAA) as the functional monomer. The binding of AFs with MIP resulted in the fluorescence burst of the MIP–QDs complexes. The concentration of AFs was quantified in accordance with the reduction in fluorescence intensity. This fluorescence sensor has been successfully used for the evaluation of AFs in non-dairy beverage samples. Similarly, as shown in Figure 4A, Chi et al. [81] proposed a fluorescence sandwich biosensor based on an MIP/porous carbon blotting layer and a CdTe/ZnS-Apt probe, which used the fluorescence property of CdTe/ZnS and functionalized it with an AFB_1_-specific aptamer. The number of recognition sites in the MIP layer was increased by combining MIP with porous carbon carriers, enhancing the sensitivity and stability of the sensor for AFB_1_ detection. The LOD was as low as 4.0 pg/mL, and the quantitative detection of spiked cooking oil samples verified its reliability and practicality for real sample analysis.

In addition to traditional QDs, carbon dots (CDs) are also commonly used as fluorescent moieties in the construction of MIP-based fluorescence sensors. The major advantages of CDs include strong luminescence and low toxicity [82,83]. As presented in Figure 4B [84], the sensor used blue carbon quantum dots (BCDs) as internal reference signals, and molecularly imprinted layers embedded with red carbon quantum dots (RCDs) and green carbon quantum dots (GCDs) were prepared using the sol–gel method to construct a B/G/RCDs/MIPs triple-emission carbon QD ratio metric fluorescence sensor. When the B/G/RCDs/MIPs are in contact with AFB_1_ or ZEN, the fluorescence intensity of GCDs (517 nm) and RCDs (619 nm) can be gradually quenched, while the fluorescence intensity of BCDs (456 nm) remains stable. Therefore, the change in fluorescence color from yellowish-green to yellow, red, violet, and finally blue enabled the naked-eye detection of AFB_1_ or ZEN. The detection limits of the method were reported as 3.2 pg/mL (AFB_1_) and 18 pg/mL (ZEN), and the spiked experiments were conducted using corn and peanut oils as the real samples.

### 3.4. SERS Sensors

At present, SERS has been extensively studied due to its advantages of high sensitivity, rapidity, non-destructiveness, and reduced photobleaching [85,86,87,88]. However, the technique lacks specificity in mycotoxin detection and is susceptible to environmental interference caused by non-specific adsorption on the SERS substrates [85]. MIPs are known as artificial receptors that are capable of specifically recognizing targets in complex matrices [89]. Therefore, the combination of SERS with MIP not only improves the selectivity of SERS, but can also enhance its sensitivity by enriching the target molecules from complex matrices [20,90,91].

**Figure 5:**
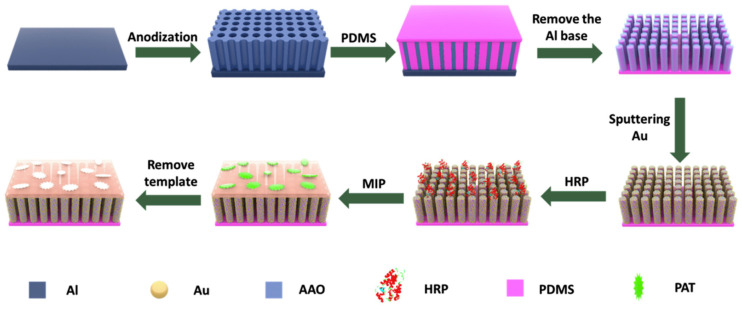
Schematic illustration of enzyme induced MIP-SERS substrate. Reproduced with permission from [93]. Copyright 2020, Elsevier.

The key to achieving high sensitivity of mycotoxins lies in the functionalization of the SERS substrate and efficiently utilizing its enhancement mechanism. Wu et al. [92] developed a molecularly imprinted Au NP-based SERS sensor for the detection of PAT in food samples. The study employed Au NPs as the SERS substrate and formed recognition sites with high selectivity for PAT by fabricating an MIP layer on their surface. Using enzymatic free radical polymerization with 4-vinylpyridine (4-VP) as the functional monomer, 1,4-diacryloylpiperazine (PDA) as the crosslinker, PAT as the template, and horseradish peroxidase (HRP) as the initiator, the sensor achieved highly sensitive and specific recognition of PAT. Under optimized conditions, the sensor showed an LOD of 5.37 × 10^−12^ M. Real sample analysis was conducted with blueberry jam, grapefruit jam, and orange juice. The project team [93] developed a novel MIP-SERS sensor (MIP-ir-Au/PDMS/AAO) by combining enzyme-initiated in situ polymerization and a polydimethylsiloxane (PDMS)/anodized aluminum oxide (AAO) substrate to enhance the SERS signal intensity and obtain reliable SERS signals. As shown in Figure 5 the PDMS/AAO substrate was first prepared by PDMS with AAO as the template, which is ideal for generating a strong SERS effect. Au NPs were sputtered on the surface of the PDMS/AAO substrate, forming a SERS substrate with the distribution of “hot spots”. HRP enzyme was immobilized on the surface of Au/PDMS/AAO substrate as an initiator for free radical polymerization to form an MIP layer, enabling the selective recognition of target molecules through the sensor. This MIP-SERS sensor demonstrated high sensitivity and selectivity for PAT, with a detection limit of 8.5 × 10^−11^ M. The study is simple and suitable for the rapid detection of PAT in food samples, without the need for complicated sample pretreatment.

### 3.5. SPR Sensors

SPR sensors are metal film-based optical sensors that use special electromagnetic waves to determine the interaction between analytes in solutions and the recognition elements immobilized on the surface of the sensor [94]. Biomolecules, such as antibodies or aptamers, are frequently employed as the recognition elements. MIPs have also been recently used to replace biomolecules in the construction of SPR sensors [95]. Integrating the advantages of both techniques for the rapid detection of mycotoxins in food is possible by introducing MIPs on the SPR platform [96]. The detection principle is as follows: when the target mycotoxins are introduced, they specifically bind to the imprinted polymer on the surface of the sensor, resulting in a change in the SPR angle, which can be detected by the sensor.

Metal nanoparticles (such as gold and silver) have proven to be the most versatile nanomaterials, benefitting from the advantages of finely tunable optical properties [97]. Akgönüllü et al. [98] used *N*-methacryloyl-L-phenylalanine (MAPA) as a functional monomer to form pre-polymerized complexes by exploiting its hydrophobic interactions with the template OTA. The MIP film was then prepared by light-initiated polymerization using 2-hydroxyethyl methacrylate as the crosslinker. The OTA-imprinted SPR sensor was finally fabricated by coating the MIP film onto the surface of a gold SPR chip modified with allyl mercaptan. The introduction of allyl mercaptan facilitates the adhesion of the MIP film to the SPR chip by forming an Au–S bond with the gold surface. The results show that the sensor, with a low detection limit of 0.028 ng/mL and high selectivity, was effective in detecting OTA in dried fig samples. Similarly, this strategy was also applied for the detection of AFB_1_ [99]. By pre-polymerizing the template AFB_1_ with the functional monomer MAPA and mixing it with the crosslinker EGDMA and AuNPs, a MIP coating can be generated on the surface of the nanoparticles through UV-light-initiated polymerization. Finally, the MIP coating was applied to the surface of the modified SPR chip, forming an MIP–SPR sensing system that selectively detects AFB_1_. The sensor demonstrated a detection limit as low as 1.04 pg/mL, with an imprinting factor of 5.91, indicating a high affinity and selectivity for AFB_1_. In addition, the sensor showed good reproducibility and storage stability when used to detect AFB_1_ in real food samples, including peanuts and corn.

In addition to noble metal nanomaterials, carbon-based nanomaterials, such as graphene and carbon nanotubes, have also been employed in the fabrication of SPR sensors. These materials allow for the introduction of various functional groups, either during synthesis or through post-synthesis modifications. Recent studies demonstrated that SPR signals can be amplified by incorporating graphic carbon nitride (g-C_3_N_4_), a low-dimensional nanomaterial with highly flexible electronic structures, good stability, and cost-effectiveness [100]. A molecularly imprinted SPR sensor for the selective detection of ZEN in rice samples was proposed by Çapar et al. [101] High-purity sulfur-doped g-C_3_N_4_/Bi_2_S_3_ (S-g-C_3_N_4_/Bi_2_S_3_) nanocomposites were synthesized through calcination treatment, and the S-g-C_3_N_4_/Bi_2_S_3_-based ZEN-imprinted SPR chip was prepared using MAGA as the monomer, EGDMA as the crosslinker, *N*,*N*′-azobisisobutyronitrile (AIBN) as the initiator, and ZEN as the template. The synthesized S-g-C_3_N_4_/Bi_2_S_3_ nanocomposite enhanced the sensitivity of the sensor. The results show that the detection limit of the ZEN-imprinted SPR chip was 0.33 ng/L, with a linear range of 1.0–10.0 ng/L.

**Table 2:** A summary of MIPs for the rapid detection of mycotoxins in foods.

Areas of Application	Mycotoxins	LODs	Linear Rages	Matrix Samples	Pre-Treatment	% Recovery	Imprinting Factor	Ref.
SPE	AFB_1_	0.1 μg/L	0.1~10 μg/L	peanut	extract with methanol/KH_2_PO_4_, filtrate	93~102%	2.19	[51]
SPE	AFB_1_	0.05 ng/mL	10~1000 ng/mL	soy sauce	extract with methanol/water, dilute with water	96%	N/A	[52]
SPE	AFs	0.05 μg/kg (AFG_2_); 0.06 μg/kg (AFG_1_); 0.06 μg/kg (AFB_2_); 0.05 μg/kg (AFB_1_);	0.1~50 μg/kg	rice, corn, wheat, peanut and soybean	extract with acetonitrile/water, filtrate, dilute with 1% Tween-20 PBS	82.6~116.7%	2.42	[53]
SPE	AFB_1_	0.4 ng/mL	5~1000 ng/mL	soy sauce, vinegar	extract with methanol, centrifuge	73~92%	1.5	[55]
SPE	AFB_1_; AFB_2_	0.0024 ng/mL (AFB_1_); 0.0004 ng/mL (AFB_2_)	0.005~0.5 ng/mL (AFB_1_); 0.001~0.1 ng/mL (AFB_2_)	corn, peanut, edible oil	extract with methanol/water, centrifuge	89~105%	N/A	[56]
SPE	AFB_1_; AFB_2_; AFG_1_; AFG_2_	0.23~0.33 μg/kg	0.1~400 μg/kg	wheat, rice, corn	extract with methanol/water, centrifuge, filtrate, dilute with phosphate buffer, refiltrate	95.3~98.5%	3.29 (AFB_1_); 2.81 (AFB_2_); 3.22 (AFG_1_); 3.00 (AFG_2_)	[62]
SPE	AFs	0.003~0.09 ng/mL	0.02~200 ng/mL (AFG_2_); 0.3~200 ng/mL (AFG_1_); 0.01~200 ng/mL (AFB_2_); 0.2~200 ng/mL (AFB_1_);	rice, corn, wheat and peanut	extract with ACN/water, centrifuge, concentrate with nitrogen stream, redissolve with ACN	85.4~105.4%	2.98	[64]
Electrochemical sensors	AFB_1_	0.52 pg/mL	1.56~31.23 pg/mL	cinnamon	N/A	98.21%	N/A	[72]
Electrochemical sensors	ZEN	0.09 ng/mL	0.25~500 ng/mL	corn meal	N/A	98.59% (50 ng/mL); 102.18% (100 ng/mL); 97.30% (250 ng/mL)	N/A	[74]
Electrochemical sensors	ZEN	0.25 ng/L	1~10 ng/L	rice	extract with EtOH/ACN, centrifuge, dilute with PBS	100%	N/A	[76]
Electrochemical sensors	ZEN	1 × 10^−4^ ng/mL	0.005~1 ng/mL	corn, rice, beer	corn and rice: extract with ACN/water, centrifuge, dilute with PBS beer: degas, dilute with PBS	96~110%	N/A	[75]
Electrochemical sensors	PT	0.66 pM	0.001~100 nm	apple juice	dilute with PBS	94.5~106.4%	15.4	[77]
Fluorescence Sensors	AFs	0.016 mg/L	4~15 μg/kg	non-dairy beverages (four almond based-, three soy based-, and three rice based-beverages)	centrifuge	99 ± 4~107 ± 5%	30.6	[80]
Fluorescence Sensors	AFB_1_	4 pg/mL	0.01~20 ng/mL	edible oil (peanut, corn, and olive)	extract with methanol/water, filtrate	91.9~102.6%	4.77	[81]
Fluorescence Sensors	AFB_1_; ZEN	3.2 Pg/mL (AFB_1_); 18 Pg/mL (ZEN)	0.01~100 ng/mL (AFB_1_); 0.03~100 ng/mL (ZEN)	corn and peanut oil	extract with methanol/water, redissolve in PBS	96.3~103.7% (AFB_1_); 99.1~102.6% (ZEN)	21.89(AFB_1_); 21.95(ZEN)	[84]
SERS Sensors	PAT	5.37 × 10^−12^ M	7 × 10^−12^~5 × 10^−8^ M	blueberry sauce, grapefruit sauce, and orange juice	extract withethyl acetate/n-hexane solution, desiccation with sodium sulfate, solvent evaporation, dilute with water	96~101%	N/A	[92]
SERS Sensors	PAT	8.5 × 10^−11^ M	5 × 10^−10^~10^−6^ M	blueberry jam, grapefruit jam and orange juice	N/A	96.43~112.83%	N/A	[93]
SPR Sensors	OTA	0.028 ng/mL	0.1~20 ng/mL	dried fig	extract with acetonitrile/water, filtrate, dilute with PBS	98 ± 2.43~100 ± 8.3%	2.85	[98]
SPR Sensors	AFB_1_	1.04 pg/mL	0.0001~10 pg/mL	ground corn, peanut	extract with methanol/water, centrifuge, dilute with PBS	96.63~105.94%	5.91	[99]
SPR Sensors	ZEN	0.33 ng/L	1~10 ng/L	rice grain	extract with ethanol/acetonitrile solution, centrifuge, dilute with PBS	N/A	N/A	[101]

## 4. Conclusions

MIT has been extensively studied in recent years for the detection of mycotoxins in food, owing to its specific recognition capability and excellent chemical stability. The effectiveness of pre-treatment methods, electrochemical, and optical sensors can be explored and optimized by preparing MIPs and incorporating them as recognition elements in various sensing platforms. This review focuses on the preparation of various MIPs and their application in the rapid detection of mycotoxins in food. As presented, MIPs have been synthesized through appropriate polymerization techniques and applied to SERS detection, electrochemical sensors, fluorescence sensors, SPR sensors, and preprocessing SPE techniques. These developments substantially improved the sensitivity, selectivity, and user-friendliness of these methods for mycotoxin detection. For example, SERS and SPR sensors use MIPs to improve the specificity of SERS by specifically identifying targets in complex matrices and enriching target molecules to increase sensitivity. The advantages of electrochemical sensors lie in their high sensitivity, low detection limit, ease of operation, and rapid test. Qualitative and quantitative analyses of mycotoxins in food can also be achieved by immobilizing MIPs as recognition elements on the electrode surface. Molecular recognition events can be converted into detectable fluorescence signals by incorporating fluorescence materials into MIPs, offering the advantages of rapid detection and high sensitivity. SPE technology, using MIPs as adsorbents, overcomes the limitations of traditional SPE, including selectivity, susceptibility to matrix interference, and low sensitivity.

Despite the remarkable progress of MIPs in mycotoxin detection, some flaws and drawbacks still exist in some studies. Therefore, the following points should be considered for conducting further research in this area:

1. The preparation conditions of MIPs play a major role in the performance of the final products. Therefore, further optimization could help to improve the performance of current MIPs. The template molecules should possess the right size, shape, and functional groups. Moreover, the functional groups should not hinder the polymerization reaction. Functional monomers should be selected as inert as possible to avoid excessive non-specific adsorption. Preparation conditions should be improved to ensure optimal adsorption performance of MIPs [102].
2. MIPs need to be evaluated for imprinting efficiency by adsorption and selectivity experiments, and performance differences need to be compared with non-imprinted polymers (NIPs).
3. The selectivity and interference resistance of MIPs in complex food matrices still need to be improved.
4. The reusability of MIPs should be further considered to address the demands of practical applications.
5. During the polymerization process, appropriate safety measures should be taken.
6. The large-scale production and commercial application of MIPs encounter cost and technical challenges.

The research of MIPs in the field of mycotoxin detection aims to address these issues, and the performance of MIPs could be further enhanced with the development of new functional materials. For example, the mechanical strength, selectivity, and adsorption capacity of MIPs can be enhanced by introducing novel materials such as MOFs [58]. The use of multi-template MIPs is also an important development to address the demand for simultaneous detection of multiple mycotoxins in food samples [39]. As the trend of intelligent data analysis continues to grow rapidly, artificial intelligence (AI) can be harnessed for the rational design of MIP materials before conducting the experiments. Currently, molecular docking simulations are commonly used to screen functional monomers for target compounds. Using AI, the precise simulation of the 3D structure of binding cavities in MIPs is achievable, enabling the rational design of MIPs based on the structure and chemical properties of the target molecules. Through pattern recognition and prediction models, early warning and risk assessment of mycotoxin contamination in food can also be conducted [103,104]. Additionally, the development of MIPs should focus more on green chemistry and sustainability by adopting environmentally friendly synthesis methods, reducing the use of hazardous solvents, and improving the reusability of MIPs [105]. Overall, MIPs have considerable potential and wide application prospects in the field of mycotoxin detection. With the development of new materials and the application of novel technologies, MIT will generally provide a more efficient, sensitive, and reliable solution for food safety detection.

## Figures and Tables

**Table 1:** Characteristics of different MIPs preparation methods and their application areas.

Preparation Method	Advantages	Shortcomings	Application Platforms
Bulk polymerization	easy-to-operate, synthetic systems conducive to the generation of more blotting sites	cumbersome post-processing steps, difficulty on removing the template	SPE, chemical sensors, drug delivery systems
Precipitation polymerization	adjustable microspheres size and shape	time-consuming, requires large amount of solvent	SPE, environmental monitoring
In-situ polymerization	preparation of imprinted materials with specific forms and functions	strict reaction conditions	biosensors, drug analysis, material preparation
Emulsion polymerization	MIP microspheres can be prepared with high specific surface area and reusability	the use of surfactants may block the binding sites	drug delivery, catalyst carriers
Suspension polymerization	MIP microspheres can be prepared in large sizes, suitable for large-scale preparation	the use of surfactants may contaminate MIPs	industrial separation, environmental pollutant treatment
Surface imprinting polymerization	most of the recognition sites are located on the outer layer of the polymer, fast mass transfer rate	strict reaction conditions	biosensors, food safety monitoring

## Data Availability

No new data were created or analyzed in this study. Data sharing is not applicable to this article.
